# Fulminant Herpes Pneumonia Postaortic Surgery with Known Ankylosing Spondylitis

**DOI:** 10.1055/s-0042-1757791

**Published:** 2022-12-20

**Authors:** Nicole Asemota, Ikenna David Ike, Aung Ye Oo, Ana Lopez-Marco

**Affiliations:** 1 Department of Cardiothoracic Surgery, St Bartholomew's Hospital, London, United Kingdom

**Keywords:** pneumonia, herpes simplex, postoperative, immunosuppression

## Abstract

Herpes simplex virus (HSV) pneumonitis is rare after cardiac surgery. A 36-year-old gentleman with ankylosing spondylitis underwent emergency surgery for a complex aortic aneurysmal disease. Preoperative treatment of aortitis with antitumor necrosis factor and steroid medication and surgical stress including cardiopulmonary bypass potentially created an immunosuppressive state and reactivation of undiagnosed HSV. Rapid HSV pneumonia ensued, culminating in fulminant organ failure and mortality. HSV pneumonia should be considered postoperatively in patients with severe respiratory distress, especially if immunocompromised.

## Introduction

Although herpes simplex virus (HSV) reactivation is not uncommon in immunosuppressed states, 1 HSV pneumonitis after cardiac surgery is rare and only sporadically reported. 1 2 3 We present a fulminant HSV pneumonitis in a young, immunosuppressed patient after surgery for a rapidly expanding aortic arch aneurysm secondary to ankylosing spondylitis.

## Case Presentation

**Fig. 1:**
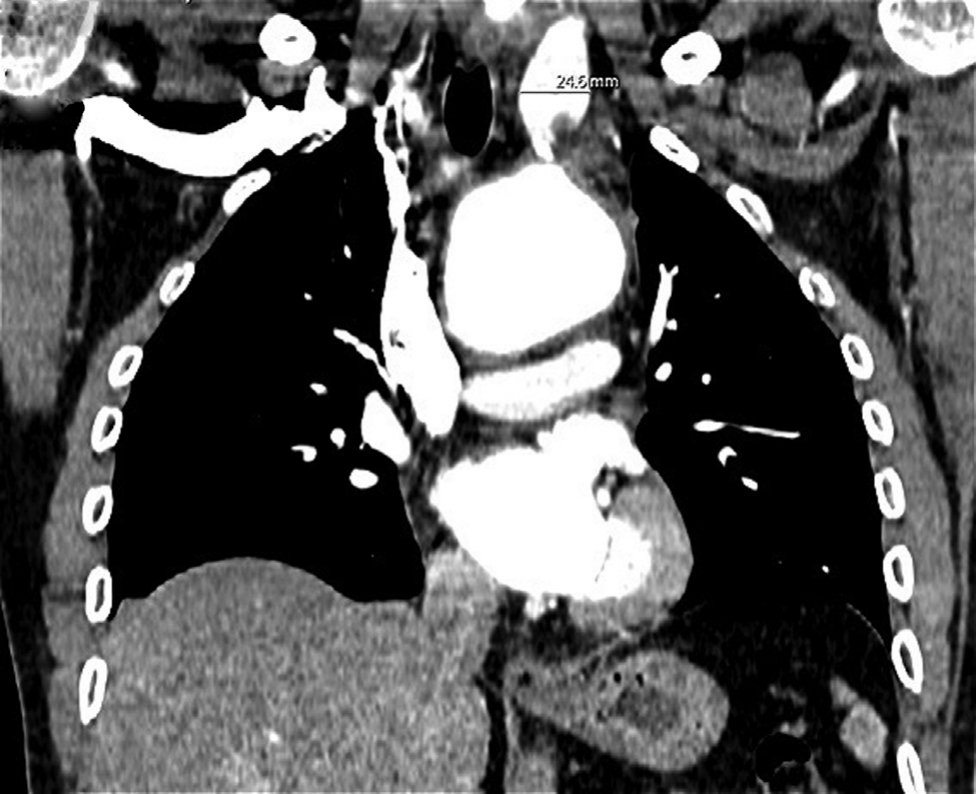
Coronal view of preoperative computed tomography scan demonstrating a 55-mm aortic arch aneurysm involving the origin of the supra-aortic trunks, with a separate aneurysm of the left common carotid (25 mm).

A 36-year-old gentleman with a background of ankylosing spondylitis (AS) was incidentally diagnosed with a 51-mm aortic arch aneurysm, involving the innominate artery (IA) and left subclavian artery (LSA), as well as a 25-mm aneurysm of the left common carotid artery (LCCA; Fig. 1 ). Surgical indication was made based on the aortic diameters, but persistently elevated inflammatory markers at the time of diagnosis compatible with acute vasculitis delayed the surgery until optimization of the inflammatory status to reduce postoperative complications.

**Fig. 2:**
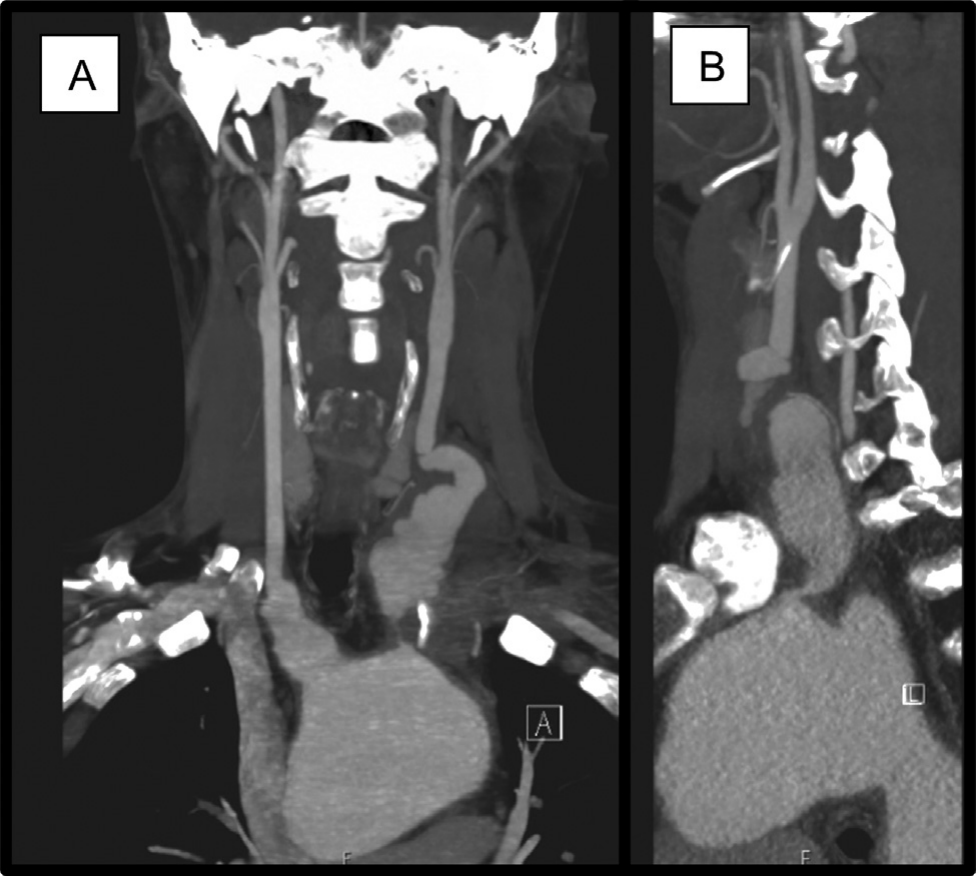
( **A** ) Coronal view and ( **B** ) sagittal view of interval preoperative computed tomography scan demonstrating expansion of left common carotid aneurysm (70 mm x 25 mm x 26 mm).

He received a course of antitumor necrosis factor (TNF) therapy (adalimumab) and intravenous methylprednisolone over 2 months until the inflammatory markers normalized. A new pulsatile mass became evident in the neck due to the rapid expansion of the LCCA aneurysm (70 mm × 25 mm × 26 mm; Fig. 2 ).

Urgent surgery was then performed and consisted of aortic arch replacement with a 30 mm × 100 mm Thoraflex frozen elephant trunk anastomosed to zone 1 of the aortic arch, with reimplantation of the IA and LCCA onto the corresponding branches of the arch graft and extra-anatomical bypass of the LSA. Surgery was performed via a ministernotomy ( **J** -shaped toward the fourth right intercostal space), and cardiopulmonary bypass (CPB) was established by cannulation of the LSA with an 8-mm Dacron graft anastomosed end to side, and the right atrium with a double stage venous cannula. Hypothermic circulatory arrest (28 °C), antegrade cerebral perfusion (via selective cannulation of IA, LCCA, and LSA), and myocardial protection with repeated administration of antegrade cold blood cardioplegia were used.

The procedure was uneventful, and he was transferred to intensive care in a stable condition. He was extubated in the early postoperative hours but developed worsening metabolic acidosis with discrete response to fluid resuscitation and bicarbonate administration. A transesophageal echocardiogram demonstrated no evidence of tamponade or cardiac impairment.

The following day, he developed fast atrial fibrillation with hemodynamic compromise, requiring both electric and pharmacological cardioversions that were ineffective.

Due to worsening metabolic acidosis and hypoxia, he was reintubated and started on continuous veno-venous hemofiltration.

**Fig. 3:**
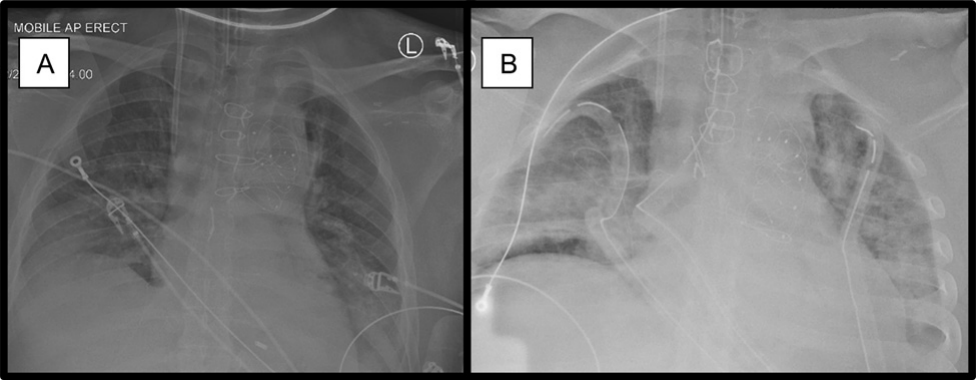
Anteroposterior erect chest X-rays: serial postoperative anteroposterior chest-X-rays taken on postoperative day 2 in the interval of 6 hours. Note the rapid progression of widespread bilateral ground-glass opacification, consisting bilateral pneumonitis.

Serial chest X-rays demonstrated progressive bilateral widespread infiltrates ( Fig. 3 ). A computed tomography scan of the chest, abdomen, and pelvis was performed, ruling out bowel ischemia.

He was taken back to the theater for reexploration with no abnormal findings.

He continued to decline with evidence of refractory ischemic hepatic failure. He was declined for extracorporeal membrane oxygenation due to worsening multiorgan failure and died shortly after.

Postmortem examination surprisingly revealed extensive bilateral bronchopneumonia due to HSV infection that had caused the progressive and refractory multiorgan failure. Histopathology analysis of the aortic tissues confirmed aortitis secondary to autoimmune disease.

## Discussion

The association between AS and cardiac disease has been widely demonstrated. The common manifestations include aortic valve regurgitation and arrhythmias, although aortitis and aneurysmal disease have also been described, rarely requiring surgical intervention. 4

Common side effects of anti-TNF therapy (used commonly in AS) include respiratory tract infections and hepatitis, 5 which are never evident preoperatively. Despite aortic aneurysm being listed as an uncommon side effect of adalimumab, 5 it is difficult to ascertain if this contributed to the rapid expansion of the aneurysm in our patient.

The relationship between immunosuppression and HSV pneumonia has been widely demonstrated. 1 HSV pneumonia is rare after cardiac surgery. 1 2 Sporadic cases are reported in both immunocompetent and immunocompromised patients, presenting with acute hypoxic deterioration (often requiring invasive ventilation), ground-glass opacification on chest radiography ( Fig. 3 ), no organisms on bacterial sputum cultures, and failure to respond to antibiotics. 1 2 3 Polymerase chain reaction analysis for HSV from bronchial aspirates is the best diagnostic investigation. 1 Many reported cases responded well to acyclovir treatment, although none of them described such fulminant multiorgan failure as in our patient. 1 2

It is unclear whether prior knowledge of latent disease would have aided in diagnosis and treatment. Viral screening and infection history are routinely conducted in heart transplant patients due to the risk of reactivations once immunosuppressed 6 ; however, due to the wide prevalence of HSV in the general population, 1 the benefit of routine preoperative screening may be minimal. Furthermore, CPB has a weakening effect on cell-mediated immunity ranging from transient to sustained suppression months after surgery. 1 2 Therefore, it can be argued that all patients should be treated as temporarily immunocompromised after bypass surgery. 1

In conclusion, we report a case with a rapidly expanding aneurysm of the aortic arch and supra-aortic trunks in the context of rheumatological disease treated with anti-TNF and steroids preoperatively. This led to an immunosuppressive state that contributed to the reactivation of unknown previous HSV infection in the immediate postoperative period causing fulminant pneumonia. HSV pneumonitis, although rare, should be considered in patients presenting with severe respiratory complications, particularly if immunocompromised. The addition of a viral screening panel should be considered for immunosuppressed patients undergoing cardiac surgery when time permits. Rapid developing and refractory hypoxia in a young, immunosuppressed patient should trigger the suspicion of fulminant viral pneumonia and consideration for early veno-venous extracorporeal membrane oxygenation.

## References

[1] Arata K Sakata R Iguro Y Toda R Watanabe S Eitsuru Y Herpes simplex viral pneumonia after coronary artery bypass grafting Jpn J Thorac Cardiovasc Surg 2003 51 04 158 159 12723587 10.1007/s11748-003-0053-0

[2] Shimokawa S Watanabe S Taira A Eizuru Y Herpes simplex virus pneumonia after cardiac surgery: report of a case Surg Today 2001 31 09 814 816 11686562 10.1007/s005950170054

[3] Breyer R H Andrews R I Mills S A Cordell A R Ahl E T Probable herpes simplex pneumonia after aortic valve replacement JAMA 1983 249 10 1319 1322 6186827

[4] Yuan S M Cardiovascular involvement of ankylosing spondylitis: report of three cases Vascular 2009 17 06 342 354 19909683 10.2310/6670.2009.00023

[5] NICE ADALIMUMAB. Drug. British National Formulary London, U.K. National Institute for Health and Care Excellence 2020

[6] Banner N R Bonser R S Clark A L UK guidelines for referral and assessment of adults for heart transplantation Heart 2011 97 18 1520 1527 21856726 10.1136/heartjnl-2011-300048

